# Influence of dietary plant fats and antioxidant supplementations on performance, apparent metabolizable energy and protein digestibility, lipid oxidation and fatty acid composition of meat in broiler chicken

**DOI:** 10.1002/vms3.212

**Published:** 2019-11-11

**Authors:** Mohammad Ali Abbasi, Shokoufe Ghazanfari, Seyed Davood Sharifi, Hassan Ahmadi Gavlighi

**Affiliations:** ^1^ Department of Animal and Poultry Sciences College of Aburaihan University of Tehran Pakdasht Tehran Iran; ^2^ Department of Food Science and Technology Faculty of Agriculture Tarbiat Modares University Tehran Iran

**Keywords:** antioxidant, digestibility, essential oils, meat quality, plant oils

## Abstract

This study aimed to evaluate the effects of dietary different antioxidants and plant oils on performance, apparent metabolizable energy and protein digestibility, meat quality and meat fatty acid composition of broiler chickens. In all, 480 male broiler chicks of 1‐day old were assigned in a completely randomized design with factorial arrangement 2 × 5 (plant oil sources [soybean and rapeseed oils] and antioxidant sources [vitamin E, Thyme, Rosemary and *Satureja* essential oils] furthermore control treatment without antioxidant). The results indicated that at 1–42 d of age, growth performance and carcass yield of birds were not influenced by dietary plant oils and antioxidant supplementations. Dietary Thyme essential oil (300 mg/kg) resulted in an increase in crude protein digestibility and birds fed on diets without antioxidant showed increase in the apparent metabolizable energy (*p* < .01). Birds receiving the combination of soybean oil with Rosemary essential oil had lowest malondialdehyde concentration in comparison to birds receiving other treatments (*p* < .05) in the drumstick meat. Also, birds receiving the combination of soybean oil with vitamin E had lowest malondialdehyde concentration in comparison to birds receiving other treatments (*p* < .05) in the breast meat. The results indicated that treatments did not influence water holding capacity of meat. Also, dietary rapeseed oil and Thyme essential oil supplementations, separately, decreased saturated fatty acid (*p* < .01) and increased unsaturated fatty acid and unsaturated to saturated fatty acids ratio (*p* < .01) of drumstick meat tissue in broiler chicken (*p* < .01). In conclusion, dietary rapeseed oil and Thyme essential oil increased in n‐3 polyunsaturated fatty acids in the drumstick meat (*p* < .01) and a combination of dietary soybean oil, Rosemary essential oil and vitamin E decreased the lipid oxidation in the meat of broiler chickens (*p* < .05).

## Graphical abstract

Dietary rapeseed oil and thymus essential oil resulted to increasing of n‐3 polyunsaturated fatty acids in the drumstick meat. Also, thymus essential oil was increased crude protein digestibility. Furthermore, the lipid oxidation was reduced by the combination of dietary soybean oil and rosemary essential oil and vitamin E in meats of broiler chicken.

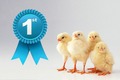

## 1 I**NTRODUCTION**

Natural antioxidants have special importance in the maintenance of high growth levels, reproduction and immunocompetence in poultry production. Insufficient intake of antioxidants, a high intake of pro‐oxidants or both may lead to oxidative stress (Gao, Lin, Wang, Song, & Jiao, 2010). Dietary polyunsaturated fatty acids (PUFA) are a group of pro‐oxidants known to increase oxidative stress in vivo in hens and chickens (Gao et al., 2010). A large proportion of PUFA in dietary plant oils increase the requirement for antioxidants (Cortinas et al., 2005). Additionally, lipid oxidation has negative effects on meat quality of broiler chickens (Brenes et al., 2008). However, an increase in the amount of n‐3 PUFAs in foods, especially docosahexaenoic (DHA) and eicosapentaenoic (EPA), may confer greater susceptibility to lipid oxidation, and oxidative deterioration adversely affects the sensory quality of products, including odours or flavours during storage (Gonzalez‐Esquerra & Leeson, 2001).

Also, animal feeds contain a range of different compounds that possess antioxidant activities including vitamin E, carotenoids, flavonoids, ascorbic acid and some other compounds (Surai, 2002). Among the numerous anti‐oxidative defence mechanisms, vitamin E is known as a potent antioxidant that prevents free radical damage to tissues. Different authors propose quite different concentrations of vitamin E as being necessary for the prevention of oxidative stress arising from the PUFA content in poultry diets. Leeson and Summers (2008) recommend 3 IU of vitamin E for each gram of added PUFA in 1 kg of feed, whereas German recommendations suggest an almost three times lower supplementation of 0.6 IU/g of PUFA.

Essential oils derived from herbs and spices have also been studied for antioxidant properties in poultry meat. Many studies have also been conducted on the effects of dietary essential oils or combinations on the performance of poultry but with varying and conflicting results***.*** While some reports (Alcicek, Bozkurt, & Cabuk, 2004) demonstrated that essential oils improved animal performance, some researchers indicated that lipid oxidation may be prevented by dietary plant extracts or essential oils in feeds for laying hens (Schiavone, Marzoni, & Romboli, 2001).

Rosemary is aromatic and medicinal plant which has been recognized to have high antioxidant activity (Carvalho, Moura, Rosa, & Meireles, 2005). The substances associated with the antioxidant activity of Rosemary are the phenolic diterpenes, such as carnasol, rosmanol, 7‐methyl‐epirosmanol, isorosmanol and carnosoic acid, and the phenolic acids, such as rosmarinic and caffeic acids. On the other hand, the major components of Rosemary volatile oil are monoterpenes such as α‐pinene, myrecene, 1,8‐cineole and borneol. These compounds possess strong antibacterial activities (Okoh, Sadimenko, & Afolayan, 2010). However, the properties of the volatile oils have been found to vary depending on extraction method used.

Thyme (*Thymus vulgaris*) is a medicinal herb that can be used as a natural alternative to antibiotics in poultry production but it also has inhibitory effects on abdominal fat traits in broiler chickens (Al‐Kassie, 2009). Al‐Kassie (2009) showed that adding 200 ppm Thyme oil to the diets of broiler chickens caused a significant reduction in the abdominal fat percentage. Volatile oil from Thyme was assessed for antibacterial and antiviral activity as inhibitors of microbial growth (Dorman & Deans, 2000). The major components of Thyme essential oil are the two predominant components, thymol and carvacrol (Lawrence & Reynolds, 1984).

*Satureja* (*Satureja hortensis* L.) is an annual, herbaceous aromatic and medicinal plant belonging to the *Lamiaceae* family. It is known as summer savory, native to southern Europe and naturalized in parts of North America (Sefidkon, Sadighzadeh, & Taymori, 2006). Its essential oil contains considerable amounts of two phenolic ketones, that is, carvacrol and thymol (Ghannadi, 2002). Different species of *Satureja* are famous for their analgesic, antiseptic, antimicrobial, antiviral, antioxidant, antiproliferative, antiprotozoal, antifungal, antidiarrheal, anti‐inflammatory, anti‐nociceptive and vasodilatory activities (Mihajilov‐Krstev, Radnovic, Kitic, Stojanovic‐Radic, & Zlatkovic, 2010).

In the present study, the effect of dietary different antioxidants (vitamin E, Thyme, Rosemary and *Satureja* essential oils) and plant oils (soybean and rapeseed) on growth performance, carcass characteristics, meat fatty acid composition, crude protein and apparent metabolizable energy digestibility and meat quality in broiler chickens will be investigated.

## 2 MATERIALS AND METHODS

### 2.1 Composition of the essential oils

The composition of the essential oils was determined using gas chromatography (GC; Agilent 6890N, Agilent Technologies, Paris, France) interfaced with mass spectroscopy (MS; Agilent 5973N, Agilent Technologies) (Table 1). The capillary column used was the HP5‐MS 5% phenyl methyl siloxan (length: 30 m; internal diameter: 0.25 mm; film thickness: 0.25 µm) and an automatic passer (Agilent 7683B; Agilent Technologies). Helium was the carrier gas at a flow rate of 1 ml/min. The column temperature was initially adjusted at 5°C (during 1 min) then increased progressively at a rate of 2°C/min to reach 300°C within 130 min. The samples were diluted in ethanol (1/10) then 1 µl was injected into GC‐MS (Bampidis et al., 2005). The components were identified by comparing their relative retention times and mass spectra with the standard data (NIST 05, Mass Spectra Library, National Institute of Standards and Technology, Gaithersburg, MD). The GCMS analyses were conducted at Institut National de la Rechercheetd’Analyses Physico‐chimiques (Sidi‐Thabet, Tunisia).

**Table 1:** Composition of essential oils of Rosemary, Thyme and *Satureja*

Rosemary Essential oil		Thyme Essential oil		*Satureja* Essential oil	
Compound^a^	(%)	Compound	(%)	Compound	(%)
α‐Pinene	24.9	Thymol	28.53	Carvacrol	50.5
β‐Pinene	1.46	Carvacrol	25.06	p‐Cymene	2.06
Borneol	0.19			α‐Thujene	0.23
p‐cymene	1.39			Myreene	0.23
Limonene	4.11			γ‐Terpenene	0.74
Cineole	15			α‐Terpinene	0.12
Camphor	5.33			α‐Pinene	0.15
Camphen	ND^2^			Limonene	0.12
β‐myrcene	ND			trans‐Sabinene hydrate	0.17
Bornyl acetate	ND			α –terpinole	0.39
α‐terpineol	ND			Thymyl acetate	ND
Verbenone	ND			Thymol	ND
				α – Humulene	ND
				β –Bisabolene	ND

2ND: compounds are not detected.

a Identification by gas chromatography coupled to mass spectroscopy (GC‐MS): National Institute of Standards and Technology (Gaithersburg, MD).

### 2.2 Animal and experimental procedures

A total number of 480 male broiler chicks of 1‐day old (Ross 308) obtained from the Mahan Breeder Company (Varamin, Tehran), were weighed and randomly assigned to 10 treatments and each group divided to four replicates with 12 male birds each in a completely randomized design with factorial arrangement 2 × 5 (plant oil sources [soybean and rapeseed oils at level of 4% of diet] and antioxidant sources [200 mg/kg vitamin E, 300 mg/kg rosemary, Thyme and *Satureja* essential oils] plus a control group that did not receive antioxidant). The basal diet was formulated to meet the nutrient requirements of the broiler chickens as recommended by Ross 308 broiler management guide (AVIAGEN, 2002) (Table 2). The experimental diets were based on corn‐soybean meal containing vegetable oil. Feed and water were provided ad libitum. Temperature and relative humidity were maintained within the optimum range. The birds were kept in 40 Cage (1 × 1.1 m) and photoperiod of 24 hr light/day was maintained during on days 1–3 of age and 23 hr light/1 hr of darkness on day 42. The ventilation rate was 0.12 m/s during the whole period. The initial house temperature was 32°C and then gradually decreased to reach 20°C at 42 days of age. All animal procedures were approved by the Animal Care Committee of the Animal Sciences Research Institute of Iran. All chicks were fed starter diets (1–10 days of age), grower diets (11–24 days of age) and finisher diets (25–42 days of age). All diets were prepared freshly every week and diets were in mash form.

**Table 2:** Composition of the experimental basal diet

	Starter (1–10) days	Grower (11–24) days	Finisher (25–42) days
Ingredient (%)			
Corn	50.8	57.0	63.9
Soybean meal, 44%	35.9	30.0	24.3
Rapeseed meal	5.00	5.00	4.00
Plant oil	4.00	4.00	4.00
Dicalcium phosphate	1.73	1.54	1.29
Oyster shell	1.12	1.06	0.99
Sodium chloride	0.25	0.25	0.25
Vitamin premix^a^	0.30	0.30	0.30
Mineral premix^b^	0.30	0.30	0.30
DL‐Methionine	0.35	0.27	0.29
L‐Threonine	0.10	0.10	0.10
L‐Lysine HCL	0.24	0.16	0.27
Total	100	100	100
Nutrient Composition (calculated)			
Crude protein (%)	21.90	20.34	18.89
Metabolizable Energy (Kcal/Kg)	2,880	2,980	3,100
Available phosphorus (%)	0.48	0.43	0.38
Calcium (%)	1.01	0.86	0.75
Methionine (%)	0.69	0.61	0.56
Methionie + Cystine (%)	1.03	0.8	0.77
Lysine (%)	1.38	1.05	0.98
Argentine (%)	1.23	1.11	1.01
Tryptophane (%)	0.28	0.20	0.18
Threonine (%)	0.90	0.87	0.78
Valine (%)	1.09	0.86	0.76
Na (%)	0.15	0.13	0.12
Cl (%)	0.21	0.21	0.23
K (%)	0.95	0.86	0.73
Crude fibre	30.50	29.30	28.10
DEB (Meq Kg^−1^)^c^	249.76	273.27	232.74

a Supplying per kg feed: all‐trans retinol acetate: 9,000 IU; cholecalciferol: 2000 IU; tocopherol acetate: 36 IU; menadione sodium bisulphate: 2 mg; thiamine mononitrate: 1.8 mg; riboflavin: 6.6 mg; pantothenic acid: 10 mg; niacin: 30 mg; choline chloride: 250 mg; biotin: 0.1 mg; folic acid: 1 mg; pyridoxine: 3.0 mg; vitamin B12: 0.015 mg; BHT: 1 mg

b Supplying per kg feed: iron: 50 mg; zinc: 85 mg; manganese: 100 mg; iodine: 1 mg; copper: 10 mg; selenium: 0.2 mg

c Represents dietary electrolyte balance as defined by dietary Na + K – Cl (in mEq/Kg of diet).

### 2.3 Performance and carcass characteristics

Feed intake and body weight were recorded during the starter period (1–10 days), grower period (11–24 days), finisher period (25–42 days) and the total experimental period (1–42 days) and then the weight gain, feed intake and feed conversion ratio (FCR) (feed/gain) were calculated.

At the 42nd day of age, two birds per pen were slaughtered and carcass, drumstick, breast, abdominal fat pad and pancreas were weighed and calculated as a percentage of live body weight.

### 2.4 Apparent metabolizable energy and digestibility of protein

Digestibility of nutrients was measured using Cr_2_O_3_ (0.3% of the grower diet) as indicator described by Petry and Rapp (1971). The digestibility study included 3‐d pre‐experimental adaptation period (20–22 days of age) followed by 3‐d collection period (23–25 days of age). During each 3‐d collection period, pooled excreta samples from respective treatment cages (*n* = 3/treatment) were collected daily. After contaminants, such as feathers and other foreign materials, were carefully removed, the excreta samples (*n* = 9/treatment) were stored in air‐tight containers at –20°C until later analysis. Feed and excreta samples were dried at 60°C for 48 hr, ground to pass through a 0.5‐mm sieve, and stored in air‐tight containers at −20°C. Gross energy was determined using an adiabatic oxygen bomb calorimeter (Parr Instrument Company, Moline, IL). All samples were analysed in duplicates. Chromic oxide concentrations in the feed and excreta were determined using the procedure described by Fenton and Fenton (1979). The digestibility coefficients (DC) of protein and AME were calculated using the following equation:

$$\text{DC}\left(\%\right)=100-\left\{100\times \left(\frac{{\text{Cr}}_{2}O_{\text{3diet}}\times {\text{nutrient}}_{\text{faecal}}}{{\text{Cr}}_{2}O_{\text{3fecal}}\times {\text{nutrient}}_{\text{diet}}}\right)\right\}$$

$$\text{AME}\left(\text{kcal/kg}\right)=\text{GE diet}-\left\{\text{GE excreta}\times \left(\frac{{\text{Cr}}_{2}O_{3\text{diet}}}{{\text{Cr}}_{2}O_{3\text{excreta}}}\right)\right\},$$

where Cr_2_O_3diet_ is the concentration of chromium in the diet (%), Cr_2_O_3excreta_ is the concentration of chromium in the excreta (%), nutrient_faecal_ is the concentration of the nutrient (also for GE) in the excreta (%), nutrient_diet_ is the concentration of the nutrient (also for GE) in the diet (%), GE_diet_ is the GE content of the diet (kcal/kg) and GE_excreta_ is the GE content of the excreta (kcal/kg).

### 2.5 Meat quality measurements

#### 2.5.1 Lipid oxidation

Malondialdehyde (MDA), the compound used as an index of lipid peroxidation, was determined by a selective third‐order derivative spectrophotometric method on the 3, 6 and 9 days of storage (Botsoglou, Florou‐Paneri, Christaki, Fletouris, & Spais, 2002). In brief, samples were homogenized in the presence of 8 ml of 5% aqueous trichloroacetic acid (Merck, Darmstadt, Germany) and 5 ml of 0.8% butylated hydroxytoluene (Sigma Chemical Co, St. Louis, MO) in hexane, and the mixture was centrifuged. The top layer was discarded, and a 2.5‐ml aliquot from the bottom layer was mixed with 1.5 ml of 0.8% aqueous 2‐thiobarbituric acid (Sigma Chemical Co, St. Louis, MO) to be further incubated at 70°C for 30 min. Following incubation, the mixture was cooled under tap water and submitted for conventional spectrophotometry (Shimadzu, Model UV‐160A, Tokyo, Japan) in the range of 400–650 nm. Third‐order derivative spectra were produced by digital differentiation of the normal spectra using a derivative wavelength difference setting of 21 nm. The concentration of MDA in analysed samples was calculated on the basis of the height of the third‐order derivative peak at 521.5 nm by referring to slope and intercept data of the computed least‐squares fit of standard calibration curve prepared using 1,1,3,3‐tetraethoxypropane (Sigma Chemical Co, St. Louis, MO).

#### 2.5.2 Water holding capacity

Water holding capacity (WHC) was determined by centrifuging, according to Bertram, Andersen, and Karlsson (2001) on samples collected 4 hr after slaughter (one sample from breast and drumstick meats per chicken). Samples weighing about 1 g were centrifuged at 4,000 x *g* for 15 min. The expressed juice was defined as the loss in weight after centrifugation at 4,000 x *g* at 15 min. The expressed juice was defined as the loss in weight after centrifuged and presented as a percentage of the initial weight of the original sample. Total moisture content was determined in duplicate according to AOAC^1^ procedures (AOAC, 2005). The WHC was calculated as the fraction of water retained by the meat (1‐(expressible juice/total moisture content)).

#### 2.5.3 Fatty acid composition analysis

Drumstick meat were analysed for their fatty acid composition following the methods of lipid extraction adapted from the procedure of Folch, Lees, and Stanley (1957) and saponification/methylation adapted from the procedure of Metcalfe, Schmitz, and Pelka (1966). Approximately 0.5 g of drumstick meat was homogenized completely in a 10‐ml mixture of chloroform and methanol (2:1). After filtration of samples through Whatman #4 filter paper (>20 to 25 μm), 2 ml of 0.88% KCl solution was added to the extract. The extract mixtures were then centrifuged at 2,000 x *g* for 30 min at room temperature. The upper phase of the extract was discarded, and the lower phase was transferred into a reaction vial and dried under nitrogen. After adding 0.5 Ml of 0.5 N NaOH in methanol to the dry samples, samples were heated for 5 min at 100°C in a heating block. Samples were cooled to room temperature; 0.5 ml of boron trifluoride in methanol was then added to the sample, after which the samples were heated an additional 5 min at 100°C and cooled to room temperature. One millilitre of hexane and 1 ml of saturated NaCl were added to samples, and samples were centrifuged at 1,500 x *g* for 5 min. After centrifugation, fatty acid methyl esters contained in the upper phase were isolated by gas chromatography on a Hewlett‐Packard 5,890 gas chromatograph. Fatty acid profiles were determined by gas–liquid chromatography (Perkin Elmer [Auto System] Gas Chrom, column:SGE [BP70X GC column] [60 m‐capillary; 0.25 μm film thickness; 0.25‐mm diameter WCOT fused‐silica], detector: flame ionization detector [FID], column temp.: ramp 1:165°C [increase with 1°C/min] [2 min isotherm], ramp 2:225°C [increase with 2°C/min] [30 min isotherm], detector temp.: 250°C, injector temp.: 250°C, elution time: 35 min., inject. amount: 2 μl, split ratio:50:1, carrier gas: Helium [He], flow rate: 1.5 ml/min). Fatty acid composition was expressed as a percentage of total fatty acids.

### 2.6 Statistical Analyses

The data were subjected to two‐way analysis of variance with plant oil and antioxidant sources as the main effects as a completely randomized design with factorial arrangement 2 × 5 using the general linear models (GLM) procedure of SAS software (SAS, 2003). Significant differences among the means were determined using Tukey's multiple‐range test at *p* < .05.

## 3 RESULTS

### 3.1 Chemical composition of the essential oils

The results obtained by GC and GC–MS analysis of the *SATUREJA*, Thyme and Rosemary essential oils are presented in Table 1. The Rosemary essential oil major component was α‐pinene (24.92%), 1,8‐Cineole (15%), limonene (4.11%) and camphor (5.33%). The Thyme essential oil major component was thymol (28.53%) and carvacrol (25.06%). The *Satureja* essential oil major component was carvacrol (50.46%).

### 3.2 Effect of dietary different antioxidants and plant oils on growth performance and carcass yield

Growth performance (feed intake, weight gain and feed conversion ratio) during the starting, growing, finishing and total periods (Table 3) and carcass yield (Table 4) were not influenced by dietary plant oils (soybean and rapeseed) and different antioxidants (Thyme, Rosemary and *Satureja* essential oils and vitamin E). According to the above study, the interaction of plant oils by antioxidant sources did not affect the growth performance and carcass yield in broiler chicken.

**Table 3:** Effects of different antioxidants and plant oils on growth performance of broilers between 1 and 42 days of age

Items	Weight gain (g/bird/d)				Feed intake (g/bird/d)				Feed conversion ratio (feed/gain)			
	1 to 10 d	11 to 24 d	25 to 42 d	1 to 42 d	1 to 10 d	11 to 24 d	25 to 42 d	1 to 42 d	1 to 10 d	11 to 24 d	25 to 42 d	1 to 42 d
Main effects												
Oil sources												
Soybean Oil	19.3	51.9	74.0	52.0	26.7	108.0	133	97	1.38	2.09	1.79	1.85
Rapeseed Oil	19.5	51.0	74.0	52.0	26.9	108.0	132	97	1.38	2.12	1.78	1.86
*SEM*	0.29	0.77	0.85	0.39	0.22	0.45	0.45	0.25	0.01	0.03	0.02	0.01
Antioxidant Sources												
Vitamin E	18.7	50.1	73.0	51.0	26.2	107.7	131	96	1.41	2.16	1.78	1.87
Rosemary EO	18.8	51.0	74.0	52.0	26.5	108.4	132	97	1.42	2.12	1.78	1.86
Thyme EO	20.3	53.0	75.0	53.0	27.3	108.0	133	97	1.35	2.03	1.76	1.81
*Satureja* EO	19.5	51.6	74.0	52.0	26.9	107.0	132	96	1.38	2.07	1.78	1.84
Without Antioxidant	20.2	51.6	73.0	52.0	27.1	109.7	134	98	1.35	2.13	1.83	1.88
*SEM*	0.46	1.21	1.35	0.62	0.35	0.72	0.71	0.4	0.02	0.05	0.03	0.02
Oil sources × Antioxidant sources												
Soybean Oil												
Vitamin E	18.2	50.0	73.0	51.0	25.4	108.2	131	96	1.40	2.18	1.77	1.87
Rosemary EO	18.5	50.0	73.0	51.0	26.7	108.3	132	97	1.44	2.17	1.81	1.89
Thyme EO	20.6	54.0	77.0	54.0	27.6	108.5	133	97	1.34	2.00	1.73	1.79
*Satureja* EO	19.9	52.0	74.0	52.0	26.9	107.0	133	97	1.39	2.02	1.80	1.84
Without Antioxidant	20.4	53.0	73.0	53.0	26.9	108.5	134	97	1.32	2.02	1.81	1.84
Rapeseed Oil												
Vitamin E	19.2	50.1	73.0	51.0	27.1	107.2	132	96	1.41	2.15	1.79	1.87
Rosemary EO	19.0	52.0	75.0	53.0	26.4	108.6	132	97	1.39	2.07	1.75	1.83
Thyme EO	19.9	52.0	74.0	52.0	27.1	107.4	132	96	1.36	2.06	1.78	1.84
*Satureja* EO	19.5	50.0	74.0	52.0	26.8	106.9	131	96	1.37	2.12	1.76	1.84
Without Antioxidant	19.9	50.0	73.0	51.0	27.2	110.6	135	98	1.37	2.21	1.84	1.91
*SEM*	0.65	1.72	1.91	0.88	0.50	1.02	1.01	0.56	0.03	0.07	0.05	0.03
*p value*												
Oil	.80	.35	.94	.51	.46	.99	.71	.94	.89	.37	.93	.52
Antioxidant	.05	.54	.78	.21	.25	.19	.11	.11	.16	.46	.75	.23
Oil × Antioxidant	.68	.46	.75	.23	.21	.58	.49	.43	.63	.35	.77	.21

EO: Essential oil

**Table 4:** Effects of different antioxidants and plant oils on carcass characteristics (% live body weight) in broiler chickens

Items	Carcass Yield%	Drumsticks%	Breast%	Abdominal fat%	Pancreas%
Main effects					
Oil sources					
Soybean Oil	77.69	26.27	36.13	1.54	0.26
Rapeseed Oil	77.57	26.42	36.49	1.66	0.25
*SEM*	0.51	0.29	0.40	0.09	0.01
Antioxidant Sources					
Vitamin E	76.72	26.13	36.28	1.69	0.24
Rosemary EO	77.68	26.63	35.98	1.69	0.27
Thyme EO	78.13	26.14	36.15	1.55	0.26
*Satureja* EO	77.81	26.53	36.69	1.57	0.25
Without Antioxidant	77.81	26.29	36.44	1.48	0.24
*SEM*	0.81	0.46	0.64	0.15	0.02
Oil sources × Antioxidant sources					
Soybean Oil					
Vitamin E	75.27	25.58	35.73	1.58	0.23
Rosemary EO	78.13	26.66	35.99	1.65	0.26
Thyme EO	79.76	26.34	36.26	1.41	0.25
*Satureja* EO	77.22	26.07	35.95	1.55	0.27
Without Antioxidant	78.05	26.72	36.71	1.52	0.27
Rapeseed Oil					
Vitamin E	78.17	26.68	36.82	1.80	0.25
Rosemary EO	77.23	26.60	35.96	1.73	0.29
Thyme EO	76.51	25.95	36.04	1.69	0.28
*Satureja* EO	78.39	26.99	37.43	1.60	0.23
Without Antioxidant	77.58	25.86	36.18	1.45	0.22
*SEM*	1.14	0.65	0.89	0.21	0.03
*p value*					
Oil	.87	.72	.53	.41	.90
Antioxidant	.77	.90	.94	.83	.78
Oil × Antioxidant	.12	.50	.75	.92	.44

EO: Essential oil

### 3.3 EFFECT of dietary different antioxidants and plant oils on apparent metabolizable energy and digestibility of protein

As shown in Table 5, different dietary antioxidants had significant effects on apparent metabolizable energy (AME) and digestibility of crude protein. The higher crude protein digestibility was observed in the dietary Thyme essential oil treatment compared to dietary vitamin E, without antioxidant and *Satureja* essential oil treatments (*p* < .01). There are not any difference in crude protein digestibility between dietary Thyme essential oil and Rosemary essential oil treatments. Without antioxidant, treatment showed highest AME when compared to the other treatments (*p* < .001). The lower AME was observed in the dietary *Satureja* essential oil treatment compared to treatments of without antioxidant and Thyme essential oil. AME and digestibility of crude protein was not influenced by the dietary plant oils. Also, the interaction of plant oils by antioxidant sources did not affect the apparent metabolizable energy and digestibility of crude protein in broiler chicken.

**Table 5:** Effects of different antioxidants and plant oils on nutrient digestibility of broilers

Items	Crude protein digestibility	AME
Main effects		
Oil sources		
Soybean Oil	66.07	2,888.3
Rapeseed Oil	67.22	2,895.9
*SEM*	0.76	12.39
Antioxidant Sources		
Vitamin E	63.29^c^	2,881.8^bc^
Rosemary EO	69.28^ab^	2,833.8^bc^
Thyme EO	69.97^a^	2,891.5^b^
*Satureja* EO	65.89^bc^	2,826.5^c^
Without Antioxidant	64.78^c^	3,026.9^a^
*SEM*	1.21	19.59
Oil sources × Antioxidant sources		
Soybean Oil		
Vitamin E	61.74	2,872.2
Rosemary EO	70.46	2,855.2
Thyme EO	70.60	2,879.9
*Satureja* EO	63.78	2,826.3
Without Antioxidant	63.75	3,008.2
Rapeseed Oil		
Vitamin E	64.84	2,891.5
Rosemary EO	68.09	2,812.4
Thyme EO	69.34	2,903.1
*Satureja* EO	67.99	2,826.7
Without Antioxidant	65.82	3,045.7
*SEM*	1.71	27.71
*p value*		
Oil	.30	.67
Antioxidant	.003	.0001
Oil × Antioxidant	.27	.64

^a–c^Means with no common superscript within each column are significantly (*p* < .05) different. EO, Essential oil; AME, Apparent Metabolizable Energy.

### 3.4 Meat quality measurements

#### 3.4.1 Effect of dietary different antioxidants and plant oils on lipid oxidation of meats

The effect of different plant oils and antioxidants on lipid oxidation drumstick and breast meats is shown in Table 6. Furthermore, the effect of different times and treatments on lipid oxidation drumstick and breast meats are shown in Table 7. Thiobarbituric acid (TBA) analysis is an efficient way to measure antioxidant activity in meat products. This analysis is an indicator of malondialdehyde (MDA), a product of oxidation; thus, the TBA value increases during the storage period. MDA is an important index for lipid peroxidation and oxidative damage caused by reactive oxygen species.

**Table 6:** Effects of different antioxidants and plant oils on lipid oxidation of drumstick and breast meat (all data points are mean malondialdehyde (MDA) concentrations) and on water holding capacity of broilers

Items	Drumstick (mg MDA/kg meat)			Breast (mg MDA/kg meat)			Water Holding Capacity (%)	
	3 Days	6 Days	9 Days	3 Days	6 Days	9 Days	Drumstick	Breast
Main effects								
Oil sources								
Soybean Oil	0.065^b^	0.190^b^	0.560^b^	0.040^b^	0.190	0.480^a^	56.2	52.6
Rapeseed Oil	0.096^a^	0.320^a^	0.820^a^	0.060^a^	0.210	0.310^b^	54.5	51.0
*SEM*	0.007	0.023	0.035	0.005	0.018	0.026	1.75	1.37
Antioxidant Sources								
Vitamin E	0.190^a^	0.350^a^	0.790^a^	0.030^bc^	0.190	0.240^d^	56.6	50.7
Rosemary EO	0.060^b^	0.290^ab^	0.560^c^	0.070^a^	0.210	0.480^ab^	54.5	49.1
Thyme EO	0.060^b^	0.230^b^	0.720^abc^	0.060^a^	0.200	0.400^bc^	57.6	53.6
*Satureja* EO	0.030^b^	0.220^b^	0.760^ab^	0.020^c^	0.200	0.330^dc^	51.6	51.5
Without Antioxidant	0.300^b^	0.190^b^	0.620^bc^	0.050^ab^	0.190	0.530^a^	56.4	53.7
*SEM*	0.011	0.037	0.055	0.008	0.029	0.042	2.64	2.06
Oil sources × Antioxidant sources								
Soybean Oil								
Vitamin E	0.110^b^	0.200	0.620^d^	0.018^d^	0.097^d^	0.250^c^	59.9	53.6
Rosemary EO	0.040^cd^	0.230	0.240^e^	0.106^a^	0.24^ab^	0.640^a^	56.8	49.1
Thyme EO	0.104^b^	0.240	0.380^e^	0.022^d^	0.290^a^	0.590^a^	53.9	56.9
*Satureja* EO	0.020^d^	0.130	0.720^cd^	0.024^d^	0.160^bcd^	0.330^bc^	51.8	49.3
Without Antioxidant	0.037^cd^	0.150	0.860^abc^	0.029^cd^	0.150^bcd^	0.580^a^	58.5	54.3
Rapeseed Oil								
Vitamin E	0.270^a^	0.500	0.960^ab^	0.060^bc^	0.290^a^	0.220^c^	53.3	47.8
Rosemary EO	0.080^bc^	0.360	0.870^abc^	0.037^cd^	0.180^abcd^	0.320^bc^	51.5	49.2
Thyme EO	0.0320^d^	0.210	1.070^a^	0.111^a^	0.120^cd^	0.200^c^	61.2	50.3
*Satureja* EO	0.040^cd^	0.310	0.810^bcd^	0.028^cd^	0.240^ab^	0.330^bc^	51.3	53.8
Without Antioxidant	0.040^cd^	0.220	0.370^e^	0.083^ab^	0.220^abc^	0.480^ab^	54.2	53.1
*SEM*	0.016	0.052	0.078	0.011	0.040	0.060	3.74	2.92
*p value*								
Oil	.006	.0009	.0001	.004	.370	.002	0.43	0.36
Antioxidant	.0001	.035	.032	.003	.970	.0007	0.51	0.53
Oil × Antioxidant	.0001	.503	.0001	.0001	.002	.014	0.36	0.33

^a–e^ Means with no common superscript within each column are significantly (*p* < .05) different. EO, Essential oil

**Table 7:** Effects of the interactions between times and treatments on lipid oxidation of drumstick and breast meat of broilers (all data points are mean malondialdehyde (MDA) concentrations)

Time	Rapeseed oil					Soybean oil					SEM	Time			SEM
	Vitamin E	Rosemary EO	Thyme EO	*Satureja* EO	Without Antioxidant	Vitamin E	Rosemary EO	Thyme EO	*Satureja* EO	Without Antioxidant		3 days	6 Days	9 days	
Drumstick (mg MDA/kg meat)															
3 days	0.114	0.047	0.104	0.025	0.037	0.277	0.084	0.032	0.047	0.042	0.055	0.081	0.260	0.695	0.017
6 days	0.205	0.230	0.249	0.013	0.152	0.509	0.361	0.212	0.313	0.229	0.055				
9 days	0.629	0.249	0.381	0.725	0.861	0.966	0.875	1.075	0.810	0.378	0.055				
*p* value															
Time		Oil*Time				Antioxidant*Time			Oil*Antioxidant*Time						
<0.0001		0.0002				0.044			<0.0001						
Breast (mg MDA/kg meat)															
3 days	0.060	0.037	0.111	0.028	0.083	0.018	0.106	0.022	0.024	0.029	0.042	0.013	0.399	0.205	0.052
6 days	0.225	0.183	0.125	0.255	0.226	0.097	0.247	0.293	0.169	0.155	0.042				
9 days	0.298	0.326	0.207	0.333	0.481	0.259	0.641	0.594	0.338	0.589	0.042				
*p *value															
Time		Oil*Time				Antioxidant*Time			Oil*Antioxidant*Time						
<0.0001		<0.0001				0.0002			0.01						

At the end of this experiment, malondialdehyde (MDA) concentration in drumstick muscle of combination of rapeseed oil and vitamin E supplemented treatment was significantly higher than the other treatments after 3 days of storage (*p* < .05). Also, lower MDA concentration was shown in the combination of soybean oil with *Satureja* essential oil and rapeseed oil with Thyme essential oil supplemented treatments compared to treatments of soybean oil with vitamin E and Thyme essential oil and rapeseed oil with vitamin E and Rosemary essential oil on day 3 day of storage in drumstick muscle (*p* < .01).

On the 6 day of storage, birds receiving soybean oil had lower MDA concentration in comparison to birds receiving rapeseed oil (*p* < .05). Bird‐fed dietary Thyme and *Satureja* essential oils had lower MDA concentration in comparison to birds fed vitamin E (*p* < .05). On the 9 day of storage, use of soybean oil with Rosemary and Thyme essential oils caused a significantly reduction of MDA content in comparison to the other treatments (*p* < .0001). Therefore, birds receiving soybean oil with Rosemary essential oil had lowest MDA concentration in comparison to birds receiving other treatments (*p* < .05).

On the breast tissue, lipid oxidation was influenced (*p* < .05) by antioxidants and plant oils interaction (*p* < .05) at 3, 6 and 9 days of storage. On the 3 day of storage, use of the soybean oil with vitamin E, Thyme and *Satureja* essential oils caused considerable decrease in oxidation breast meat in comparison to the combination of soybean oil with Rosemary essential oil and rapeseed oil with vitamin E, Thyme essential oil and without antioxidant treatments (*p* < .0001). On the 6 day of storage, bird‐fed dietary soybean oil with vitamin E was shown lower MDA content in comparison to birds receiving soybean oil with Rosemary and Thyme and rapeseed oil with vitamin E, *Satureja* essential oil and without antioxidant treatments (*p* < .01). On the 9 day of storage, the use of dietary soybean oil with vitamin E and *Satureja* essential oil and rapeseed oil with vitamin E, rosemary, Thyme and *Satureja* essential oils were decreased MDA concentration in breast tissue (*p* < .05) in comparison to other treatments. Therefore, birds receiving soybean oil with vitamin E had lowest MDA concentration in comparison to birds receiving other treatments (*p* < .05).

#### 3.4.2 Effect of dietary different antioxidants and plant oils on water holding capacity

The effect of different antioxidants and plant oils on water holding capacity is shown in Table 6. Diets with different antioxidants (rosemary, Thyme and *Satureja* essential oils and vitamin E) and plant oils (soybean and rapeseed) had no marked effect on water holding capacity in drumstick and breast meat. Also, the interaction of plant oils and essential oils had no significant effect (*p* ˃ .05) on water holding capacity.

#### 3.4.3 Effect of dietary different antioxidants and plant oils on fatty acids composition

The results of fatty acid composition of the drumstick meat are presented in Table 8. Results showed that the addition of rapeseed oil to the diets modified the fatty acid composition of drumstick muscle by reducing (*p* < .01) saturated fatty acid and increasing (*p* < .01) unsaturated fatty acid in drumstick meat. Also, use of the rapeseed oil to the diets caused significant increase in unsaturated to saturated fatty acids ratio in the drumstick meat (*p* < .01).

**Table 8:** Effects of different antioxidants and plant oils on fatty acid profile of drumstick meat in broilers (%)

Items		C14:0	C14:1	C16:0	C16:1	C18:0	C18:1	C18:2	C18:3	C20:1	C20:2	C22:0	C22:6	U	S	U/S ratio
Main effects																
Oil source																
Soybean Oil		0.482^a^	0.042	22.13^a^	3.72^a^	2.28^b^	42.55^b^	25.66^a^	0.15	2.39^b^	0.136	0.32	0.125^b^	74.77^b^	25.20^a^	2.98^b^
Rapeseed Oil		0.436^b^	0.036	19.12^b^	3.24^b^	2.77^a^	50.58^a^	19.81^b^	0.16	3.09^a^	0.138	0.35	0.170^a^	77.23^a^	22.70^b^	3.41^a^
*SEM*		0.005	0.003	0.25	0.07	0.07	0.71	0.66	0.007	0.05	0.004	0.01	0.012	0.30	0.30	0.05
Antioxidant Sources																
Vitamin E		0.472^a^	0.040^ab^	20.43^a^	3.54^b^	2.77^a^	45.65	23.47	0.22^a^	2.69^b^	0.157^a^	0.37	0.172^a^	75.95^ab^	24.04^ab^	3.17^ab^
Rosemary EO		0.467^a^	0.050^a^	21.40^a^	4.08^a^	1.96^b^	44.42	24.38	0.14^b^	2.56^b^	0.137^ab^	0.28	0.107^b^	75.88^ab^	24.11^ab^	3.19^ab^
Thyme EO		0.420^b^	0.027^c^	19.21^b^	2.89^c^	2.76^a^	49.30	21.45	0.15^b^	3.06^a^	0.125^b^	0.40	0.175^a^	77.18^a^	22.79^b^	3.40^a^
*Satureja* EO		0.480^a^	0.032^bc^	21.49^a^	3.50^b^	2.85^a^	46.35	21.88	0.14^b^	2.61^b^	0.140^ab^	0.34	0.150^ab^	74.82^b^	25.16^a^	3.00^b^
Without Antioxidant		0.455^a^	0.040^abc^	20.76^a^	3.39^b^	2.29^b^	47.07	22.51	0.14^b^	2.77^b^	0.122^b^	0.30	0.130^ab^	76.17^ab^	23.81^ab^	3.20^ab^
*SEM*		0.008	0.005	0.41	0.12	0.12	1.12	1.04	0.01	0.079	0.007	0.02	0.0192	0.48	0.48	0.08
Oil sources × Antioxidant sources																
Soybean Oil																
Vitamin E		0.490^abc^	0.050	22.01	3.89	2.26^cde^	40.72	27.37^a^	0.16^b^	2.39	0.150^ab^	0.35^bc^	0.135^bcd^	74.87	25.1	2.98
Rosemary EO		0.495^ab^	0.055	23.38	4.16	1.98^de^	41.92	25.18^ab^	0.15^b^	2.18	0.130^bc^	0.27^cd^	0.100^d^	73.88	26.1	2.84
Thyme EO		0.460^bcd^	0.030	20.67	3.30	2.31^cde^	47.10	22.80^bc^	0.14^bc^	2.66	0.110^cd^	0.29^bcd^	0.110^cd^	76.25	23.7	3.22
*Satureja* EO		0.510^a^	0.030	23.39	3.58	2.76^abc^	41.52	25.35^ab^	0.15^b^	2.11	0.140^ab^	0.32^bcd^	0.120^cd^	73.01	26.9	2.71
Without Antioxidant		0.455^cd^	0.045	21.17	3.64	2.10^de^	41.47	27.62^a^	0.18^b^	2.61	0.145^ab^	0.38^bc^	0.16^abcd^	75.88	24.1	3.14
Rapeseed Oil																
Vitamin E		0.455^cd^	0.040	18.86	3.18	3.27^a^	50.58	19.57^cd^	0.29^a^	2.99	0.160^a^	0.39^ab^	0.210^ab^	77.03	22.9	3.36
Rosemary EO		0.440^d^	0.045	19.42	3.99	1.93^e^	46.93	23.58^abc^	0.14^bc^	2.94	0.145^ab^	0.30^bcd^	0.115^cd^	77.89	22.1	3.54
Thyme EO		0.380^e^	0.025	17.74	2.48	3.21^a^	51.50	20.10^cd^	0.16^b^	3.46	0.140^ab^	0.50^a^	0.240^a^	78.11	21.8	3.58
*Satureja* EO		0.450^d^	0.035	19.59	3.42	2.94^ab^	51.19	18.41^d^	0.14^bc^	3.12	0.145^ab^	0.36^bc^	0.185^abc^	76.64	23.3	3.28
Without Antioxidant		0.455^cd^	0.035	20.35	3.13	2.49^bcd^	52.68	17.40^d^	0.09^c^	2.93	0.100^d^	0.22^d^	0.100^d^	76.47	23.5	3.26
*SEM*		0.012	0.007	0.57	0.17	0.17	1.59	1.48	0.01	0.11	0.009	0.04	0.027	0.68	0.68	0.12
*p value*																
Oil		.0001	.228	.0001	.0004	.003	.0001	.0001	.52	.0001	.74	.21	.016	<.0001	<.0001	.0001
Antioxidant		.0008	.05	.005	.0001	.0001	.066	.306	.0002	.002	.009	.06	.1	.041	.0395	.049
Oil × Antioxidant		.0406	.83	.087	.259	.026	.149	.045	.0001	.072	.01	.004	.026	.12	.12	.17

^a–e^Means with no common superscript within each column are significantly (*p* < .05) different. EO, Essential oil; U, Total Unsaturated, S, Total Saturated.

Also, use of the Thyme essential oil to the diets caused significant increase in unsaturated fatty acid and unsaturated to saturated fatty acids ratio and decrease saturated fatty acids compared with *Satureja* essential oil in the drumstick meat (*p* < .05). Diets with vitamin E, *SATUREJA* and Rosemary essential oils and without antioxidant treatments had no marked effect on unsaturated fatty acid, unsaturated to saturated fatty acids ratio and saturated fatty acids.

## 4 DISCUSSION

### 4.1 Effect of dietary different antioxidants and plant oils on growth performance

Mathlouthi et al. (2012) determined that the there was no differences in weight gain from 1 to 42 d of age when broiler chickens were fed Rosemary essential oil than the other treatments. Basmacioglu, Tokusoglu, and Ergul (2004) determined that the use of Rosemary oil at 150–300 mg/kg concentration in the broiler diet improved the live weight gain value (*p* < .05) in comparison to the control group. Kassie (2008) confirmed that the anise and Rosemary oils significantly improved live weight gain and the feed conversion value (*p* < .05). These results may have derived from the positive effects of aromatic herbs and their volatile oils in the digestive system, where they can improve the activity of enzymes that help in the digestion of feed (Jamroz & Kamel, 2002). The improvements of broiler chicken growth performance could be partly explained by the increase in the apparent digestibility of dietary protein and the prececal digestive capacity in general, which increase the intestinal availability of nutrients for absorption and consequently lead animals to grow faster (Mohammadi, Ghazanfari, & AdibMoradi, 2014;Windisch, Schedle, Plitzner, & Kroismaryr, 2008).

Plant oils have commonly been used as energy sources in diets of broiler chicks. Advantages of utilizing oils in poultry diet include decrease in feed dust, increase in absorption and hydrolysis of lipoproteins supplying the essential fatty acids and improved absorption of vitamin A, vitamin E and Ca. However, the chemical structures of fats and oils are extremely variable and therefore the metabolizability and also response of the animal to type of oil may be affected by its source. The important factor affecting the amount of fat metabolizable energy is their digestibility, and it is dependent on the age of birds, the length of carbon chain and the degree of saturation of fatty acids (Leeson & Summers, 2008). Moreover, Leeson and Summers (2008) reported that the optimum ratio of unsaturated to saturated fatty acids for maximizing fat digestibility and the metabolizable energy value of fat is around 3 to 1.

Therefore, absence of positive effect of antioxidants in some experiments may be due to using a smaller dose which was insufficient to produce its effect on poultry. Also, the conflicting results of the effects of plant oils on performance traits could be due to different levels of incorporation of dietary fatty acid in the diets and the sources of n‐3 PUFA. It seems that the level and source of n‐3 PUFA affect the performance in chicks. Furthermore, results in terms of functional traits are contrary to the results of some experiments in which the use of antioxidant compounds and plant oils improved the functional traits. But in our experiment, these effects were not observed.

### 4.2 Effect of dietary different antioxidants and plant oils on carcass characteristics

These findings are in agreement with the results of Ocak et al. (2008) who found no differences in carcass and organs weight of broilers fed a diet containing 2% Thyme powder. Similar results were observed by Hernandez, Madrid, Garcia, Orengo, and Megias (2004) who found no difference in weight of organs of broiler chickens fed diets containing an extract from Thyme and oregano. Also, supplementation of broiler feed with *Satureja* cannot significantly alter the carcass, abdominal fat and breast and thigh muscle percentages. Alcicek et al. (2004) examined the effects of essential oil on hot and cold carcass yield values after 42 d of feeding. The carcass yield value was reported to be increased in the group receiving essential oil. Also, in the present experiment, interaction of plant oils and antioxidant sources had no significant effect (*p* ˃ .05) on carcass traits.

The results of experiment indicate that abdominal fat of broilers were no affected by antioxidant treatments and it was contrary to the results of some experiments which abdominal fat was the affected of antioxidant compounds. The reduction in the abdominal fat traits caused by essential oils supplementation may have been attributable to the saponins in essential oils, which have inhibitory effects on lipogenesis, but in our experiment, these effects were not observed. Absence of positive effect of antioxidants in some experiments may be due to using a smaller dose which was insufficient to produce its effect on poultry.

### 4.3 Effect of dietary different antioxidants and plant oils on apparent metabolizable energy and digestibility of protein

Herbs and spices have traditionally been used to stimulate the production of endogenous secretions in the small intestinal mucosa, pancreas and liver, and thus aid digestion. Mansoub (2010) reported that the carvacrol in herbs and essential oils has stimulatory influence on pancreatic secretions by increasing the secretions of digestive enzymes, which more amounts of nutrients like amino acids can be digested and absorbed from the intestine and thereby improve some of the carcass characteristics. Herbs and phytogenic products could control and limit the growth and colonization of numerous pathogenic and non‐pathogenic species of bacteria in chicks guts. This may lead to a greater efficiency in the utilization of food, resulting in enhanced growth and improved feed efficiency. Also, there are studies that dietary essential oils addition could improve the digestion process. Lee et al. (2003) reported that cinnamaldehyde in diet had a role in the digestion process. In another study in which thymol was used, pancreatic total and activities of trypsin and total lipase activity were significantly greater in 50 mg of thymol than those in the control diet. It has been reported that feeding essential oil, extracted from herbs, improved the secretion of pancreatic digestive enzymes in broiler chickens. A mixture of carvacrol, cinnamaldehyde and capsaicin used as feed additive for broilers is shown to enhance activities of pancreatic trypsin and α‐amylase in tissue, as well as in the jejuna chyme content (Jang, Ko, Kang, & Lee, 2007).

Plant oils, usually rich in polyunsaturated fatty acids, are excellent and economical sources of energy routinely added in diets of fast‐growing broiler chickens. In chickens, oil and fat digestion occurs mainly in the duodenal segment of the intestines. Subsequent to its emulsification by conjugated bile salts, PUFA of dietary oils are hydrolyzed by pancreatic lipases into mixtures consisting essentially of 2‐monoacylglycerides and free fatty acids (FFA). The linkage of monoglycerides and long‐chain unsaturated fatty acids to conjugated bile salts promptly forms micelles. Micelles play key roles in solubilizing fatty acids with low polarity and fat‐soluble vitamins and leading to their absorption across the intestinal epithelium (Leeson & Summers, 2008).

### 4.4 Meat quality measurements

#### 4.4.1 Effect of dietary different antioxidants and plant oils on lipid oxidation of meats

Because of high proportion of PUFA, poultry meat is more susceptible to oxidative processes, specifically lipid oxidation, than beef or pork. Unfortunately, plant oils rich in PUFA are highly susceptible to oxidative deterioration. The products of lipid oxidation can decrease the nutrient content of the feed by reacting with proteins, lipids and fat‐soluble vitamins, which may even form toxic products that can adversely affect broiler performance and health (Smet et al., 2008). Therefore, incorporation of dietary antioxidants, such as vitamin E and essential oils in poultry feed, has been implemented to achieve optimal growth performance, reproduction and meat quality.

Yesilbag, Eren, Agel, Kovanlikaya, and Balci (2011) reported that groups treated with Rosemary or Rosemary volatile oil had the lowest MDA values among the groups; the MDA values were especially low on the 5th d of storage and concluded that Rosemary and its volatile oil has more antioxidant activity than α‐tocopherol acetate over a long storage period. Basmacioglu et al. (2004) used diets with added α‐tocopherol acetate, Rosemary and Thyme, together and separately, and the meat samples were exposed to higher oxidation when the storage time was extended; the breast meat was found to be more durable than the thigh meat after 15 d of storage. They also reported that rosemary, Thyme oil and α‐tocopherol acetate prevented meat from oxidation equally effectively during the early storage period, but Rosemary and Thyme oil were better at preventing the meat from oxidation than α‐tocopherol acetate over a longer storage period. The plants and their essential oils are rich in phenol compounds like polyphenols that might inhibit free radical formation. Thus, the inhibition of lipid oxidation might be related to the phenolic compounds in essential oils.

Sahin, Sahin, and Yaralioglu (2001) reported that vitamin E inhibit production of free radicals by blocking lipid peroxidation. Morrissey, Brandon, Buckley, Sheehy, and Frigg (1997) reported that dietary supplementation of chicken diets with α‐tocopherol increased concentration while markedly decreasing MDA concentration. Vitamin E is well accepted as the first line of defence against lipid peroxidation. By its free radical quenching activity, vitamin E breaks chain propagation and thus terminates free radical attack at an early stage.

#### 4.4.2 Effect of dietary different antioxidants and plant oils on water holding capacity

Dransfield and Sosnicki (1999) reported that water holding capacity is related to pH value, and observed that the lower final pH resulted in a decrease in water holding capacity. In postmortem muscle, the substrates glycogen, glucose and glucose‐6‐phosphate are converted to lactate through anaerobic glycolysis. Lactate accumulation and the release of protons from adenosine triphosphate hydrolysis in postmortem muscle induce a pH decline. A rapid pH decline may induce protein denaturation, resulting in decreased juiciness and less intense (or pale) muscle coloration. Poultry meat with low pH has been associated with low water holding capacity, which results in increased cook‐loss and drip loss (Qiao, Fletcher, Smith, & Northcutt, 2001). This finding is consistent with the observations of Mahmoodi Bardzardi, Ghazanfari, Salehi, and Sharifi (2014), who reported that essential oils of diets had no effect on water holding capacity.

#### 4.4.3 Effect of dietary different antioxidants and plant oils on fatty acids composition

The lipids in poultry exhibit a higher degree of unsaturation compared with red meat because of a relatively high content of phospholipids. The degree of unsaturation of phospholipids in subcellular membranes is an important factor in the determination of oxidative stability of meats (Coetzee & Hoffman, 2001). In relation to the character of the auto‐oxidation process, the effect of antioxidants is more significant the sooner they are applied. The ideal situation is when fats are protected immediately after the slaughter of animals. Such protection can be achieved by feeding animals with feed containing antioxidants (Coetzee & Hoffman, 2001). Feeding of poultry with a higher level of natural dietary antioxidants provides the poultry industry with a simple method for improvement of the oxidative stability, sensory quality, shelf life and acceptability of poultry meats. Vitamin E and essential oils belong to substances with significant anti‐oxidative activity.

The fatty acid profile of meat could be altered by adding plant oils to the feed for broilers (Zelenka, Jarosova, & Schneiderova, 2008). Generally, the concentrations of n‐3 PUFA increased, whereas n‐6 PUFA tended to decrease in meat lipids in response to dietary n‐3 PUFA. The PUFA are the most sensitive fractions to oxidation processes and lipid oxidation in meat are one of the reasons for quality degradation during storage. Enhancement of unsaturated fatty acids in drumstick lipids would result from diminution of fatty acid oxidation in drumstick, especially at the birds to the received rapeseed oil. This antioxidant activity of vitamin E was supported in the present study; the linolenic fatty acid concentration in drumstick meat was significantly greater than to other treatments. It was thought that the antioxidant activity of vitamin E blocked lipid peroxidation of drumstick lipids, especially linolenic fatty acid. For this reason, linolenic fatty acid in the drumstick in birds fed diets supplemented with vitamin E increased compared to other groups birds and also, linolenic fatty acid in the drumstick in birds fed diets with rapeseed oil and without antioxidants, had significant decrease (*p* < .05) compared to other treatments.

Botsoglou et al. (2002) determined that α‐tocopherol acetate and Thyme oil have nearly similar antioxidant activities, while α‐tocopherol acetate was sometimes found to be more efficient. Vitamin E is well accepted as the first line of defence against lipid peroxidation. By its free radical quenching activity, vitamin E breaks chain propagation and thus terminates free radical attack at an early stage (McDowell, 1989). Also, medicinal plants and essential oils may have the potential to improve the fatty acids due to the plant phenolic compounds (e.g. polyphenols) and organic acids because they exerted the antioxidant potential and that might inhibit free radical formation and consequently prevent the oxidation of PUFA (Jang et al., 2008). The anti‐hyperlipidaemic activity due to the phenolic acids of medicinal plants might be attributed in the improvement of the fatty acid content of broiler meat through the lipid homeostasis and fatty acid synthase enzyme (Lin, Hsu, & Yin, 2013). Bolukbasi, Erhan, and Ozkan (2006) reported that the addition of Thyme oil to the broiler feed led to a significant reduction in the saturated (SFA) and polyunsaturated fatty acid (PUFA) concentrations of the leg and breast tissues. Consistent to that, several researchers have suggested that it would be possible to modify the fatty acid contents in poultry meat through dietary manipulation of natural plant materials (Ahmed et al., 2015;Sarker, Kim, & Yang, 2009). This finding suggests that fatty acid profile of meat could be altered by adding plant oils to the feed for broilers and antioxidants can extend the shelf life and improve the quality of meat products.

## 5 CONCLUSION

Results of the present study indicated that feeding broiler chickens with rapeseed oil resulted in increasing unsaturated fatty acids and decreasing saturated fatty acids as compared to soybean oil in the drumstick meat. Furthermore, the feeding broiler chickens with Thyme essential oil resulted in increasing unsaturated to saturated fatty acids ratio as compared to other treatments in the drumstick meat. Therefore, dietary rapeseed oil and Thyme essential oil resulted to increasing in n‐3 polyunsaturated fatty acids in the drumstick meat. Also, Thyme essential oil was increased crude protein digestibility. Furthermore, the lipid oxidation was reduced by the combination of dietary soybean oil and Rosemary essential oil and vitamin E in meats of broiler chicken.

## CONFLICTS OF INTEREST

The authors hereby certify that they have no conflict of interest.

## ETHICAL STATEMENT

The authors confirm that the ethical policies of the journal, as noted on the journal's author guidelines page, have been adhered to. No ethical approval was required as this is a case report with no original research data. Informed consent was obtained from the client for the publication of this case report.

## ENDNOTE

1 Association of Official Analytical Chemists.

## References

[vms3212-bib-0001] Ahmed, S. T. , Islam, M. M. , Bostami, A. B. M. R. , Mun, H. S. , Kim, Y. , & Yang, C. J. (2015). Meat composition, fatty acid profile and oxidative stability of meat from broilers supplemented with pomegranate (*PunicaGranatum*L.) byproducts. Food Chemistry, 188, 481–488. 10.1016/j.foodchem.2015.04.140 26041221

[vms3212-bib-0002] Alcicek, A. , Bozkurt, M. , & Cabuk, M. (2004). The effects of a mixture of herbal essential oils, an organic acid or a probiotic on broiler performance. South African Journal of Animal Science, 34, 217–222.

[vms3212-bib-0003] Al‐Kassie, G. A. M. (2009). Influence of two plant extracts derived from thyme and cinnamon on broiler performance. Pakistan Veterinary Journal, 29, 169–173.

[vms3212-bib-0004] Bampidis, V. A. , Christodoulou, V. , Florou‐Paneri, P. , Christaki, E. , Spais, A. B. , & Chatzopoulou, P. S. (2005). Effect of dietary dried oregano leaves supplementation on performance and carcass characteristics of growing lambs. Animal Feed Science and Technology, 121, 285–295. 10.1016/j.anifeedsci.2005.02.002

[vms3212-bib-0005] Basmacioglu, H. , Tokusoglu, O. , & Ergul, M. (2004). The effect of oregano and rosemary essential oils or alpha‐tocopheryl acetate on performance and lipid oxidation of meat enriched with n‐3 PUFA’s in broilers. South African Journal of Animal Science, 34, 197–208.

[vms3212-bib-0006] Bertram, H. C. , Andersen, H. J. , & Karlsson, A. H. (2001). Comparative study of low‐field NMR relaxation measurements and two traditional methods in the determination of water holding capacity of pork. Meat Science, 57, 125–132. 10.1016/S0309-1740(00)00080-2 22061354

[vms3212-bib-0007] Bolukbasi, S. C. , Erhan, M. K. , & Ozkan, A. (2006). Effect of dietary thyme oil and vitamin E on growth, lipid oxidation, meat fatty acid composition and serum lipoproteins of broilers. South African Journal of Animal Science, 36, 189–196.

[vms3212-bib-0008] Botsoglou, N. A. , Florou‐Paneri, E. , Christaki, D. , Fletouris, J. , & Spais, A. B. (2002). Effect of dietary oregano essential oil on performance of chickens and on iron‐induced lipid oxidation of breast thigh and abdominal fat tissues. British Poultry Science, 43, 223–230. 10.1080/00071660120121436 12047086

[vms3212-bib-0009] Brenes, A. , Viveros, A. , Goni, I. , Centeno, C. , Sayago‐ Ayerdy, S. G. , Arija, I. , & Saura‐Calixto, F. (2008). Effect of grape pomace concentrate and vitamin E on digestibility of polyphenols and antioxidant activity in chickens. Poultry Science, 87, 307–316. 10.3382/ps.2007-00297 18212374

[vms3212-bib-0010] Carvalho, R. N. , Moura, L. S. , Rosa, P. T. V. , & Meireles, M. A. A. (2005). Supercritical fluid extraction from rosemary (*Rosmarinusofficinalis*): Kinetic data, extract’s global yield, composition and antioxidant activity. The Journal of Supercritical Fluids, 35, 197–204. 10.1016/j.supflu.2005.01.009

[vms3212-bib-0011] Coetzee, G. J. M. , & Hoffman, L. C. (2001). Effect of dietary vitamin E on the performance of broilers and quality of broiler meat during refrigerated and frozen storage. South African Journal of Animal Science, 31, 161–175. 10.4314/sajas.v31i3.3799

[vms3212-bib-0012] Cortinas, L. , Barroeta, A. C. , Villaverde, C. , Galobart, J. , Guardiola, F. , & Baucells, M. D. (2005). Influence of the dietary polyunsaturation level on chicken meat quality: Lipid oxidation. Poultry Science, 84, 48–55. 10.1093/ps/84.1.48 15685941

[vms3212-bib-0013] Dorman, H. J. D. , & Deans, S. G. (2000). Antimicrobial agents from plants: Antimicrobial activity of plant volatile oils. Journal of Applied Microbiology, 88, 308–316. 10736000 10.1046/j.1365-2672.2000.00969.x

[vms3212-bib-0014] Dransfield, E. , & Sosnicki, A. A. (1999). Relationship between muscle growth and poultry meat quality. Poultry Science, 78, 743–746. 10.1093/ps/78.5.743 10228972

[vms3212-bib-0015] Fenton, T. W. , & Fenton, M. (1979). An improved procedure for the determination of chromic oxide in feed and feces. Canadian Journal of Animal Science, 59, 631–634. 10.4141/cjas79-081

[vms3212-bib-0016] Folch, J. , Lees, M. , & Stanley, G. H. S. (1957). A simple method for the isolation and purification of total lipids from animal tissues. Journal of Biological Chemistry, 224, 497–509. 13428781

[vms3212-bib-0017] Gao, J. , Lin, H. , Wang, X. J. , Song, Z. G. , & Jiao, H. C. (2010). Vitamin E supplementation alleviates the oxidative stress induced by dexamethasone treatment and improves meat quality in broiler chickens. Poultry Science, 89, 318–327. 10.3382/ps.2009-00216 20075285

[vms3212-bib-0018] Ghannadi, A. (2002). Composition of the essential oil of Saturejahortensis L. seeds from Iran. Journal of Essential Oil Research, 14, 35–36.

[vms3212-bib-0019] Gonzalez‐Esquerra, R. , & Leeson, S. (2001). Alternatives for enrichment of eggs and chicken meat with omega‐3 fatty acids. Canadian Journal of Animal Science, 81, 295–305. 10.4141/A00-092

[vms3212-bib-0020] Hernandez, F. , Madrid, J. , Garcia, V. , Orengo, J. , & Megias, M. D. (2004). Influence of two plant extracts on broiler performance, digestibility, and digestive organ size. Poultry Science, 83, 169–174. 10.1093/ps/83.2.169 14979566

[vms3212-bib-0021] Jamroz, D. , & Kamel, C. (2002). Plant extracts enhance broiler performance. Journal of Animal Science, 80(suppl.1), 4. (Abstr.).

[vms3212-bib-0022] Jang, A. , Lin, X. D. , Shin, M. H. , Lee, B. D. , Lee, S. K. , Lee, J. H. , & Jo, C. (2008). Antioxidative potential of raw breast meat from broiler chicks fed a dietary medicinal herb extract mix. Poultry Science, 87, 2382–2389. 10.3382/ps.2007-00506 18931191

[vms3212-bib-0023] Jang, I. S. , Ko, Y. H. , Kang, S. Y. , & Lee, C. Y. (2007). Effect of commercial essential oils on growth performance, digestive enzyme activity and intestinal microflora population in broiler chickens. Animal Feed Science and Technology, 134, 304–315.

[vms3212-bib-0024] Kassie, G. A. M. (2008). The effect of anise and rosemary on broiler performance. International Journal of Poultry Science, 7, 243–245. 10.3923/ijps.2008.243.245

[vms3212-bib-0025] Lawrence, B. M. , & Reynolds, R. J. (1984). The botanical and chemical aspects of oregano. Perfumere and Flavorist, 9, 41–51.

[vms3212-bib-0026] Lee, K. W. , Everts, H. , Kappert, H. J. , Frehner, M. , Losa, R. , & Beymen, A. C. (2003). Effects of dietary essential oil components on growth performance, digestive enzymes and lipid metabolism in female broiler chickens. British Poultry Science, 44, 450–457. 10.1080/0007166031000085508 12964629

[vms3212-bib-0027] Leeson, S. , & Summers, J. D. (2008). Commercial Poultry Nutrition, 4th ed Nottingham, UK: Nottingham University Press.

[vms3212-bib-0028] Lin, M. , Hsu, P. , & Yin, M. (2013). Protective effects of *houttuyniacordata*aqueous extract in mice consuming a high saturated fat diet. Food & Function, 4, 322–327. 10.1039/C2FO30228D 23165792

[vms3212-bib-0029] Mahmoodi Bardzardi, M. , Ghazanfari, S. , Salehi, A. , & Sharifi, S. D. (2014). Effect of dietary myrtle essential oil on iron‐induced lipid oxidation of breast, thigh and abdominal fat tissues and serum biochemical parameters in broiler chickens. European Poultry Science, 78, 1–11.

[vms3212-bib-0030] Mansoub, N. H. (2010). Effect of probiotic bacteria utilization on serum cholesterol and triglycrides contents and performance of broiler chickens. Globaal Veterinaria, 5, 184–186.

[vms3212-bib-0031] Mathlouthi, N. , Bouzaienne, T. , Oueslati, I. , Recoquillay, F. , Hamdi, M. , Urdaci, M. , & Bergaou, R. (2012). Use of rosemary, oregano, and a commercial blend of essential oils in broiler chickens: In vitro antimicrobial activities and effects on growth performance. Journal of Animal Science, 90, 813–823. 22064737 10.2527/jas.2010-3646

[vms3212-bib-0032] McDowell, L. R. (1989). Vitamins in animal nutrition: comparative aspects to human nutrition, in Vitamin E. In L. R. Mc Dowell (Ed.), London : Academic Publisher, 93‐131.

[vms3212-bib-0033] Metcalfe, L. D. , Schmitz, A. A. , & Pelka, J. R. (1966). Rapid preparation of fatty acid esters from lipids for gas chromatographic analysis. Analytical Chemistry, 38, 514–515. 10.1021/ac60235a044

[vms3212-bib-0034] Mihajilov‐Krstev, T. , Radnovic, D. , Kitic, D. , Stojanovic‐Radic, Z. , & Zlatkovic, B. (2010). Antimicrobial activity of *Saturejahortensis L*. essential oil against pathogenic microbial strains. Archives of Biological Science Belgrade, 62, 159–166.

[vms3212-bib-0035] Mohammadi, Z. , Ghazanfari, S. , & AdibMoradi, M. (2014). Effect of supplementing clove essential oil to the diet on microflora population, intestinal morphology, blood parameters and performance of broilers. European Poultry Science, 78, 1–11.

[vms3212-bib-0036] Morrissey, P. A. , Brandon, S. , Buckley, D. J. , Sheehy, P. J. A. , & Frigg, M. (1997). Tissue content of α‐tocopherol and oxidative stability of broilers receiving dietary α‐tocopherol acetate supplement for various periods post‐slaughter. British Poultry Science, 38, 84–88. 10.1080/00071669708417945 9088618

[vms3212-bib-0037] Ocak, N. , Erener, F. , Burak, A. K. , Sungu, M. , Altop, A. , & Ozmen, A. (2008). Performance of broilers fed diets supplemented with dry peppermint (*Menthapiperita L*.) or thyme (*Thymus vulgaris L*.) leaves as growth promoter source. Czech Journal of Animal Science, 53(4), 169–175.

[vms3212-bib-0038] Okoh, O. O. , Sadimenko, A. P. , & Afolayan, A. J. (2010). Comoarative evaluation of the antibacterial activities of the essential oils of *Rosmarinusofficinalis L.* obtained by hydrodistillation and solvent free microwave extraction methods. Food Chemistry, 120, 308–312.

[vms3212-bib-0039] Petry, H. , & Rapp, W. (1971). Zur Problematik der Chromoxidebestimmung in Verdauungsversuchen. Zeitschrift Für Tierphysiologie Tierernährung Und Futtermittelkunde, 27, 181–189. 5577412

[vms3212-bib-0040] Qiao, M. , Fletcher, D. L. , Smith, D. P. , & Northcutt, J. K. (2001). The effect of broiler breast meat color on pH, moisture, water holding capacity, and emulsification capacity. Poultry Science, 80, 676–680. 10.1093/ps/80.5.676 11372721

[vms3212-bib-0041] Sahin, K. , Sahin, N. , & Yaralioglu, S. (2001). Effects of vitamin C and vitamin E on lipid peroxidation, blood serum metabolites, and mineral concentrations of laying hens reared at high ambient temperature. Biological Trace Element Research, 85, 35–45. 10.1385/BTER:85:1:35 11881797

[vms3212-bib-0042] Sarker, M. , Kim, G. , & Yang, C. J. (2009). Effect of green tea (*Camellia sinensis*) and mixed probiotics on meat oxidation, fatty acids profile and cholesterol in broilers. (pp. 16–21).In: Proceedings of 55th International Congress of Meat Science and TechnologyCopenhagen, Denmark

[vms3212-bib-0043] SAS (2003). SAS/STAT User’s Guide, Release 8.02 Ed. (Cary, NC, USA, SAS Institute Inc.).

[vms3212-bib-0044] Schiavone, A. , Marzoni, M. , & Romboli, I. (2001). Influence of vitamin E, rosemary (RosmarinusofficinalisL.) and orange (Citrus aurantiumL.) extracts on lipid stability of raw meat of muscovy duck (CairinamoschatadomesticaL.) fed high polyunsaturated fatty acid diets. In: Proc. XVthEuropeanSymp. on Quality of Poultry Meat, September 2001, Kuşadası, Turkey. 139.

[vms3212-bib-0045] Sefidkon, F. , Sadighzadeh, L. , & Taymori, M. (2006). The study of antimicrobial effects of essential oils of Satureiahortensis. Journal of Medicinal Plants, 23, 174–182.

[vms3212-bib-0046] Smet, K. , Raes, K. , Huyghebaert, G. , Haak, L. , Arnouts, S. , & De Smet, S. (2008). Lipid and protein oxidation of broiler meat as influenced by dietary natural antioxidant supplementation. Poultry Science, 87, 1682–1688. 10.3382/ps.2007-00384 18648067

[vms3212-bib-0047] Surai, P. F. (2002). Natural Antioxidants in Aivian Nutrition and Reproduction (pp. 669–676). Nottingham: Nottingham University Press.

[vms3212-bib-0048] Windisch, W. , Schedle, K. , Plitzner, C. , & Kroismaryr, A. (2008). Use of phytogenic products as feed additives for swine and poultry. Journal of Animal Science, 86, 140–148. 10.2527/jas.2007-0459 18073277

[vms3212-bib-0049] Yesilbag, D. , Eren, M. , Agel, H. , Kovanlikaya, A. , & Balci, F. (2011). Effects of dietary rosemary, rosemary volatile oil and vitamin E on broiler performance, meat quality and serum SOD activity. British Poultry Science, 52, 472–482. 10.1080/00071668.2011.599026 21919575

[vms3212-bib-0050] Zelenka, J. , Jarosova, A. , & Schneiderova, D. (2008). Influence of n‐3 and n‐6 polyunsaturated fatty acids on sensory characteristics of chicken meat. Czech Journal of Animal Science, 53, 299–305. 10.17221/356-CJAS

